# Microbial Pattern of Neonatal Sepsis in the Neonatal Intensive Care Unit of dr. Ramelan Navy Central Hospital

**DOI:** 10.1155/2024/6264980

**Published:** 2024-05-28

**Authors:** Stefani Miranda, Aminuddin Harahap, Dominicus Husada, Fara Nayo Faramarisa

**Affiliations:** ^1^ Department of Child Health Faculty of Medicine Hang Tuah University/dr. Ramelan Navy Central Hospital, Jalan Gadung No. 1, Surabaya, East Java 60244, Indonesia; ^2^ Department of Child Health dr. Ramelan Navy Central Hospital, Jalan Gadung No. 1, Surabaya East Java 60244, Indonesia; ^3^ Department of Child Health Faculty of Medicine Universitas Airlangga/Dr. Soetomo Academic General Hospital, Jalan Prof. Dr. Moestopo 6-8, Surabaya East Java 60286, Indonesia; ^4^ Department of Clinical Microbiology dr. Ramelan Navy Central Hospital, Jalan Gadung No. 1, Surabaya East Java 60244, Indonesia

## Abstract

**Background:**

The morbidity and mortality rates from neonatal sepsis remain high. However, there is limited information about the microbial pattern of neonatal sepsis in Indonesia. Microbial patterns can give an overview of the hygiene of an environment and act as a determinant for choosing definitive antibiotic treatment in neonatal sepsis patients. The organisms that cause neonatal sepsis differ from unit to unit and from time to time within the same unit.

**Objectives:**

This study is aimed at discovering the microbial pattern of neonatal sepsis in the Neonatal Intensive Care Unit (NICU), dr. Ramelan Navy Central Hospital, in 2021–2022.

**Methods:**

This is a retrospective, cross-sectional study that takes secondary data from the NICU and clinical microbiology department of dr. Ramelan Navy Central Hospital. Data that met the inclusion and exclusion criteria available between January 1, 2021, and December 31, 2022, were collected. Patients whose blood cultures were positive for bacterial growth and diagnosed with sepsis were selected as the study sample.

**Results:**

Out of 174 samples, 93 (53.4%) were found positive for bacterial infection and diagnosed as neonatal sepsis. Gram-negative isolates (96.8%) were predominant. Sixty-point-two percent of *Klebsiella pneumoniae XDR*, 19.4% of *Klebsiella pneumoniae ESBL*, and 8.6% of *Burkholderia cepacia XDR* were identified. The gram-positive isolates found in this study were only 3 samples (3.2%). Two-point-one percent of *MRSA* and 1.1% of *Staphylococcus haemolyticus MDR* were identified.

**Conclusion:**

The most common microorganisms causing neonatal sepsis in our NICU were gram-negative bacteria, particularly *Klebsiella pneumoniae XDR*. Following the recommended infection control procedures, practicing good hand hygiene, and having access to basic supplies and equipment are important to prevent and reduce the incidence of sepsis.

## 1. Background

Newborns with infections are known to have poor outcomes [1]. Infections in newborns are still common, especially systemic infections known as neonatal sepsis. Neonates with sepsis require intensive care in the NICU due to respiratory distress and hemodynamic instability [2]. A retrospective cohort study by Vizcarra-Jiménez et al. stated that 18.4% of neonates died while being treated in the NICU [3]. Survival rates for neonatal sepsis patients requiring intensive care are reported to be low, according to a prospective observational study by Flannery et al. [4]. This phenomenon is a problem for clinicians, especially those working in the NICU.

The morbidity and mortality of neonatal sepsis are still high. Neonatal sepsis is said to be a burden on the state because of the high incidence rate, especially in developing countries [5], like Indonesia. This high incidence rate increases the length of stay in the hospital and provides poor neurodevelopmental outcomes [6]. Odabasi and Bulbul, in their review, stated that the incidence of neonatal sepsis was 1–5 cases per 1000 live births globally. Two million six hundred thousand newborns died each year, of which 15% were caused by sepsis [1]. An observational descriptive study conducted by Nurrosyida et al. at the NICU of Dr. Soetomo General Hospital stated that 90 out of 289 cases of neonatal death were caused by sepsis [7]. A similar study has never been conducted at dr. Ramelan Navy Central Hospital.

Newborns are prone to infections caused by an underdeveloped immune system [8], especially premature babies, due to a lack of maternal antibody transfer. Maternal IgG is transferred across the placenta at the 32^nd^ week of gestation [9]. The skin surface and mucosa, as a physical barrier of the immune system, will encounter a potential pathogen for the first time. If the physical barrier maturation process is incomplete and can be penetrated by a pathogen, it will form a local infection, which can develop into a systemic infection [10]. Pathogens that cause neonatal sepsis range from bacteria and fungi to viruses. A review conducted by Ershad et al. stated that *Staphylococcus aureus*, *Coagulase-negative staphylococci*, *Streptococcus pneumoniae*, *Group B Streptococcus*, *Escherichia coli*, *Salmonella typhi*, *Coxsackie virus*, *Adenovirus*, *Rhinovirus*, *Herpes simplex virus*, and *Candida* are the most common causes of neonatal sepsis [11].

Blood culture remains the gold standard for diagnosing neonatal sepsis. False-negative results are often obtained from the blood culture due to various reasons, including the timing of blood sampling and the amount of blood taken being inappropriate, the use of antibiotics for the mother, the administration of antibiotics before blood sampling, the number of bacteria in the blood being inadequate, or a short time of bacteremia in neonates [1, 8]. We wanted to know the microbial pattern in the NICU of dr. Ramelan Navy Central Hospital to determine the causes of sepsis in neonates, so appropriate immediate management can be given besides waiting for culture results. The results of this study are expected to provide an overview of the microbial pattern of neonatal sepsis in the NICU of dr. Ramelan Navy Central Hospital in particular and, in general, in developing countries like Indonesia.

## 2. Methods

### 2.1. Study Overview

This is a retrospective, cross-sectional study that takes secondary data from the NICU and clinical microbiology department of dr. Ramelan Navy Central Hospital, Surabaya, Indonesia. This hospital is a tertiary naval hospital that provides standardized services in accordance with the Ministry of Health of the Republic of Indonesia for all of society and acts as the highest referral hospital in East Java. This hospital has three NICUs, which are divided into the central NICU, emergency room NICU, and infectious disease NICU. The total capacity of the NICU at dr. Ramelan Navy Central Hospital is 18 beds. Alcohol-based hand sanitizers were provided in each bed, and alcohol-based hand rub solutions were provided at hand-wash sinks with sufficient clean disposable tissue paper for hand-drying. Surveillance cultures were only used when an outbreak was suspected; they were not routinely undertaken. Blood cultures were taken from any infant who presented with clinical signs and symptoms of sepsis. Altered responsiveness, feeding difficulties, abnormal heart rate, hypoxia, temperature abnormality, and signs of respiratory distress were considered clinical manifestations of neonatal sepsis, according to the NICE guideline [12]. In this hospital, a general policy of using one culture bottle exclusively for newborns with at least 1 mL of blood was implemented. Within two hours of being collected, the sample was delivered to the clinical microbiology department. Routine microbiology tests, such as organism identification and antimicrobial susceptibility testing (AST), were performed by the clinical microbiology department. We use blood agar plates (BAP) and MacConkey agar as the medium for blood culture. Blood cultures were performed and incubated automatically using the VITEK-2 compact system and the AST procedure. The AST of pathogens was performed in accordance with the recommendations of the Clinical and Laboratory Standards Institute.

### 2.2. Population and Samples

#### 2.2.1. Inclusion Criteria

The inclusion criteria for this study were neonates treated in the NICU of dr. Ramelan Navy Central Hospital, whose blood was taken for culture examination and diagnosed with sepsis.

#### 2.2.2. Exclusion Criteria

The exclusion criteria for this study were neonates whose blood cultures were negative and showed fungal growth.

#### 2.2.3. Definitions

Neonate is an infant under 28 days of age.

Neonatal sepsis is the combination of clinical signs of sepsis in a neonate with the microbiological detection of a pathogen [13].

Early-onset sepsis is the occurrence of sepsis at or before the first 7 days of life [13].

Late-onset sepsis is the occurrence of sepsis after the first 7 days of life [13].

### 2.3. Sample Size Determination

This study's sample size was determined using a single population proportion formula. The neonatal sepsis proportion was derived from a study conducted in Surabaya, East Java, which reported a proportion of 10.6% [14]. Under the assumption of a 5% margin of error and a 95% confidence interval, the final sample size for this study was 146 neonates.

### 2.4. Sampling Techniques

The sampling technique used was total sampling, which was taken consecutively. All neonates admitted to our NICU between January 1, 2021, and December 31, 2022, who met the inclusion and exclusion criteria, were included because we could not meet the sample size already calculated in this study.

### 2.5. Data Collection and Management

Data collection began by obtaining the list of blood culture examination requests for neonatal patients from the clinical microbiology department's data entry. Then, from a number of secondary data sources obtained, patients whose blood cultures were positive for bacterial growth and diagnosed with sepsis were selected. The diagnosis of sepsis was searched manually through the patient's electronic medical records. The attending physician was responsible for making the diagnosis of sepsis. Neonatal sepsis patients with an International Classification of Diseases- (ICD-) 10 code of P360 to P368 were selected for this study. All medical records obtained were checked to ensure the fulfillment of the inclusion criteria. The patients' names, addresses, and hospital numbers were not recorded. The hospital numbers were only written in the master log record.

Two-year data were compiled for isolated microorganisms' growth from the blood culture and characteristics of the patients, including gender, onset of sepsis, birth weight, gestational age, Lubchenco curve, mode of delivery, history of premature rupture of membranes for more than 18 hours, type of amniotic fluid, history of antibiotic use during delivery, congenital malformations, admission type, length of hospital stay, Apgar score at 5 minutes, history of using respiratory support devices, history of being placed on a vascular access, and outcome. The data was then transferred to the Statistical Package for the Social Sciences (SPSS) database.

### 2.6. Data Analysis

Once the data were available, descriptive analysis was then performed with SPSS version 29 (SPSS Inc., Chicago, IL). Descriptive analysis was performed by finding the frequency distributions of the patient's characteristics and the isolated microorganism growth from the blood culture.

### 2.7. Ethical Clearance

This study received ethical approval from the research ethics committee of dr. Ramelan Navy Central Hospital (11/EC/KEP/2023). A waiver of informed consent was granted.

## 3. Results

### 3.1. Searching for Medical Records

**Figure 1:**
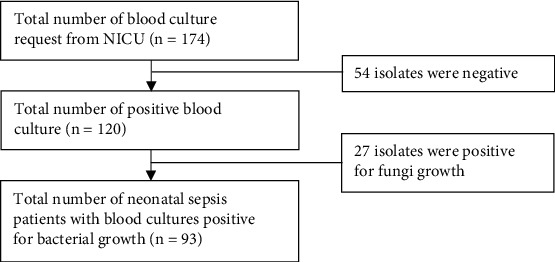
The medical record searching flow.

This study looked at 174 electronic medical records from the NICU of dr. Ramelan Navy Central Hospital, Indonesia. Finally, 93 neonates who were diagnosed with sepsis by each attending physician and confirmed with a blood culture examination were included in this study. Fifty-four neonates had negative blood culture results, and 27 neonates had positive blood culture results for fungi growth.  Figure 1 depicts the process of searching for medical records.

### 3.2. Patient Characteristics

**Figure 2:**
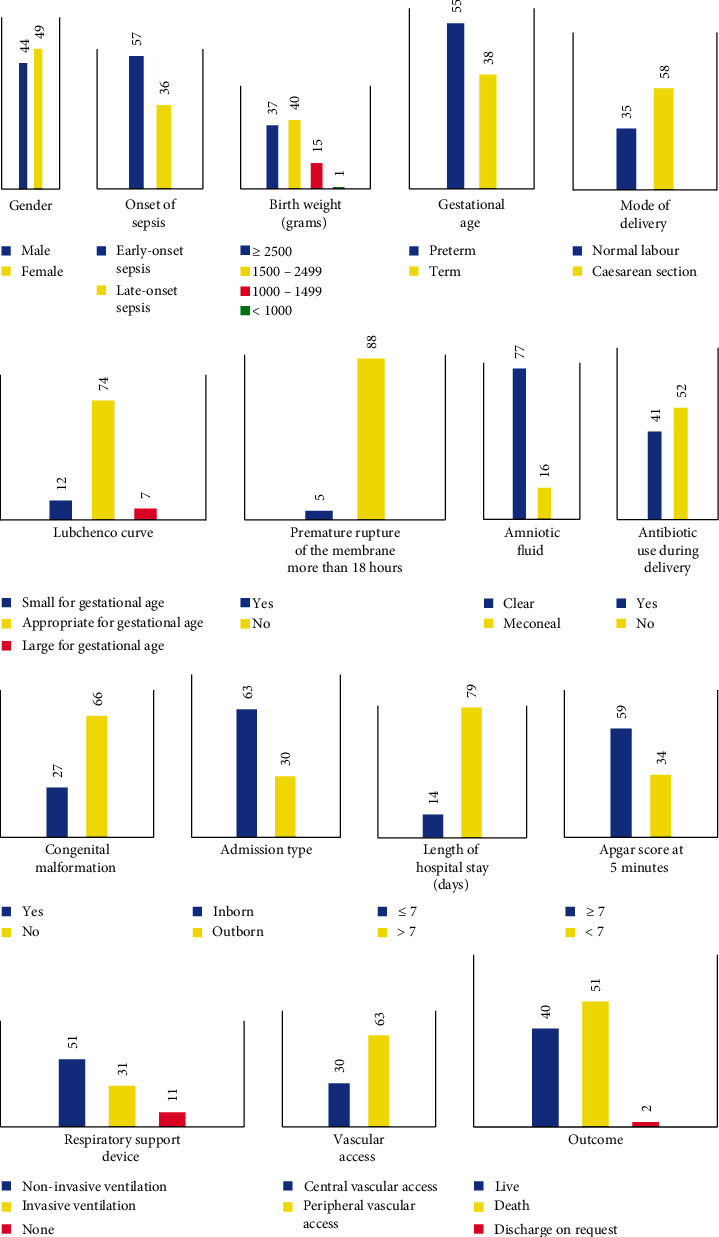
Demographic and clinical characteristics of the study population.

For 2 years, there were 174 requests for blood culture examinations made from the NICU of dr. Ramelan Navy Central Hospital. This study included 53.4% of the total examination requests.  Figure 2 shows the characteristics of the studied neonates. Most of the studied patients in this hospital were female, weighted between 1500 and 2499 g, and diagnosed with early-onset sepsis.

### 3.3. The Microbial Pattern

**Figure 3:**
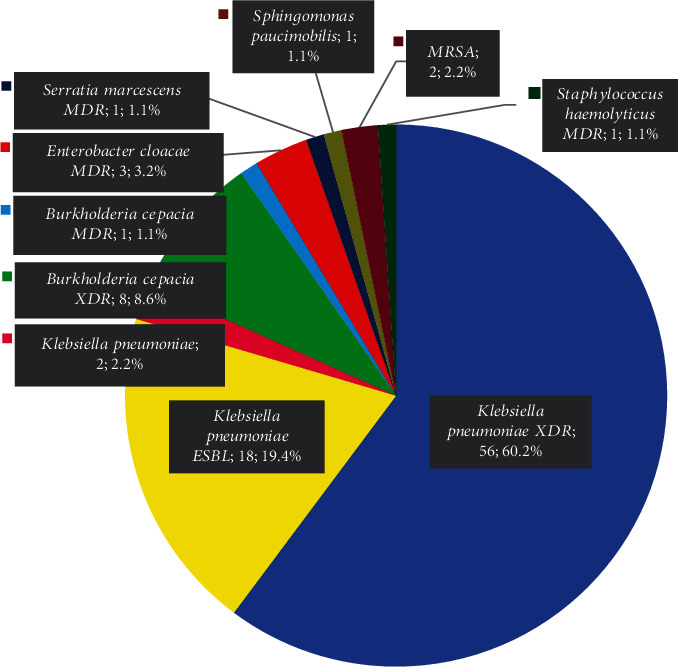
The proportion of the growth of microorganisms in the blood culture.

There were 93 neonatal sepsis patients who showed positive culture results for bacterial growth in the blood. Gram-negative isolates (90; 96.8%) were more prevalent in this study. The most common bacteria were from the genus *Klebsiella* (Figure 3).

## 4. Discussion

Neonatal sepsis is a leading cause of death and morbidity. Septicaemia is typically characterized by bacteraemia as well as a constellation of signs and symptoms caused by microorganisms or their toxic products in the circulatory system. According to the NICE guideline, clinical manifestations of neonatal sepsis include altered responsiveness, feeding difficulties, abnormal heart rate, hypoxia, temperature abnormality, and signs of respiratory distress. Signs and symptoms of sepsis, accompanied by a positive blood culture result, can lead to the diagnosis of neonatal sepsis [12]. The ICD-10 code P36 is used to code neonatal sepsis [15]. Sepsis is a significant cause of newborn death, particularly in very low-birth-weight and premature infants. According to the World Health Organization (WHO), 1.6 million newborn babies die worldwide each year as a result of neonatal infections. Despite recent advances in neonatal intensive care and current strategies to treat neonatal sepsis, mortality rates for babies born to mothers who received intrapartum antibiotic prophylaxis (IAP) for *Group B Streptococcus* have remained stable for more than three decades [16].

In this study, gram-negative bacteria were the main cause of septicaemia. This is in accordance with studies conducted by Pooja et al., Kurma et al., Govindaraju et al., and Kamalakannan, which stated that gram-negative rods are the main cause of neonatal sepsis [17], especially *Klebsiella pneumoniae* [18–20]. Contrary to this, Li et al. found gram-positive rods as the main cause of neonatal sepsis in their study [21]. The probable reason is that newborns most probably acquire these gram-negative organisms from the maternal genital tract. The importance of both vertical transmission from the mother and postnatal acquisition of infection from the environment has been suggested in the literature for the pathogenesis of neonatal sepsis [19]. This is consistent with our findings, which found that the prevalence of early-onset sepsis (EOS) was higher than that of late-onset sepsis (LOS).

Nosocomial infection is a major public health concern, particularly in NICUs in developing countries [22]. In this study, 63 of 93 newborns (67.7%) were inborn patients who stayed in our hospital for more than 7 days (84.9%). Approximately two-thirds of the pathogens identified in a study conducted by Liu et al. were responsible for hospital-acquired late-onset sepsis (HALOS). Gram-negative bacteria were responsible for approximately 60% of HALOS [22]. *Klebsiella pneumoniae* was the most common gram-negative bacteria found in South Asia and Egypt. In contrast, the most common pathogen for LOS in Western countries was *Coagulase-negative Staphylococcus* (CoNS) [23]. The predominance of gram-negative bacteria in developing countries can be attributed to a lack of standard infection control practices. Inadequate hand hygiene; a lack of essential equipment and supplies such as sinks, running water, and disposables; and overcrowding and understaffing have all been identified as major contributors to nosocomial infections caused by gram-negative bacteria [22]. The majority of routine swab culture results from the hospital staff and ward were positive for *Klebsiella pneumoniae*, indicating that nosocomial infection played a role in the incidence of sepsis in this study.

Most of the newborns in our study were of low birth weight (LBW; 43%), which is in accordance with a study conducted by Kurma et al. and Agnche et al. [19, 24]. LBW babies only had a terminal cytotoxic component value such as C3 and C3b of about 10% or less of the content of their mother and difficulty activating the complement through lectin and alternative ways. Consequently, the result is a complement dysfunction, such as a chemotaxis function, opsonization, and killing of the pathogens through the membrane attack complement. In LBW babies, there is also a lack of molecular reactants such as C-reactive protein (CRP), inhibitor protein, A amyloid protein, and several coagulation proteins, all of which serve to increase resistance to infection. Babies with LBW have a large number of neutrophils in their circulatory pool, but their bone marrow reserves are only about 20%, resulting in severe neutropenia in the state of sepsis [25].

Female newborns were predominant in this study compared to male newborns (52.7% vs. 47.3%). This result was in line with a study conducted by Worku et al., which showed that female newborns were the dominant population [26]. But it was contrary to studies conducted by Kurma et al., Agnche et al., and Guo et al. (2019), which showed otherwise [19, 24, 27]. Among the study population, 55 (59.1%) of the newborns were born preterm. Govindaraju et al., Kurma et al., and Guo et al., through their studies, also reported that most of their study population were preterm newborns [18, 19, 27]. However, studies conducted by Agnche et al. and Worku et al. were dominated by full-term newborns [24, 26]. This occurred because preterm babies had a limited capacity to increase neutrophil production in response to infection and neutrophil dysfunction [25].

The impaired neutrophil function is reported to be apparent when it is less than 30 weeks of gestation. L-Selectin expression in newborns with more than 32 weeks of gestation is approximately 40% that of adults. This sum is equivalent to the term infant. Newborns < 32 weeks have a low sFcRIII concentration and increase faster when it is 33–36 weeks of gestation. Opsonization and phagocytosis are initiated by sFcRIII. A decrease in opsonization and complement protein deficiency also occurs, whereas both of these are the main components of nonspecific immunity, which is the first line of defense against microorganisms in neonates. It makes the newborn susceptible to infection, which can often progress to a severe infection. Furthermore, IgG transfers begin at 12 weeks of gestation and peak at 400 mg/dL at 32 weeks. Physical barriers such as the skin, the mucosa membrane, and chemicals that are antibacterial or inhibit the adhesion of bacteria to the host began to mature around 32–34 weeks of gestation and accelerated after birth [25].

Most of the patient's mothers did not experience premature rupture of the membranes (PRoM) for more than 18 hours (94.6%). This contrasts with a study conducted by Salsabila et al., in which the majority of the study population had a maternal history of PRoM [28]. Although ascending infection and PRoM may necessitate caesarean delivery, neonates who develop EOS after birth may not have been exposed to the mother's microbiome during vaginal delivery and may have acquired the pathogen elsewhere [29]. In this study, 58 newborns (62.4%) were delivered via caesarean section. A retrospective study in South Africa also reported that 63.9% of their newborns were born via caesarean section [30]. In contrast, another study conducted in Ethiopia found that most of their study population was born via spontaneous vaginal delivery [26].

Our study population showed that 77 of the newborns (82.8%) were born with clear amniotic fluid, without congenital malformations found in 71% of the cases. Those results were in accordance with a study conducted by Hayun, in which 85.5% of their newborns were born with clear amniotic fluid [25]. Fecal contamination of amniotic fluid was found to have a correlation with the high prevalence of gram-negative bacteria infection in Ethiopia [26]. However, a study conducted by Lloyd et al. found that 13.7% of their study population had patent ductus arteriosus (PDA) as their comorbid [30]. More than half of the newborns (55.9%) in this study were not exposed to antibiotics during delivery, and 63 of them (67.7%) were placed on peripheral vascular access.

Studies have demonstrated that neonates with a low Apgar score have an increased risk of various interventional procedures and poor adaptation to extrauterine life, increasing their susceptibility to infection [28, 31]. But in our study, 59 out of 93 newborns (63.4%) had a high Apgar score (≥7) at 5 minutes. Most of our newborns were placed on noninvasive ventilation to support their breathing (54.8%). Despite the nature of a high Apgar score as the majority characteristic in this study, 51 (54.8%) of the newborns were dead at the end of the disease. Gram-negative bacteria as the etiology of infection may explain this phenomenon. Neonates with a gram-negative infection have a higher risk of adverse outcomes and death compared to those with a gram-positive infection or no sepsis [30].

*Klebsiella* species have previously been reported to be a dominant species in neonatal sepsis in low- and middle-income countries (LMICs) [29]. *Klebsiella* spp. can reside in the hospital environment, the gastrointestinal tract, the birth canal, and on the surfaces of medical devices. These species are a major public health concern, particularly in health care settings where antimicrobial agents are scarce. In this study, newborns infected with *Klebsiella* spp. could have gotten the bacteria from their mother either vertically (EOS) or horizontally (LOS) [26]. However, the high proportion of *Klebsiella* spp. in our study may not be explained by vaginal colonization alone. Molecular characterization may be required to confirm the source of the infection. The current study found a high prevalence of bacteria causing neonatal sepsis as well as an alarmingly high level of antibiotic resistance in the isolates. Most of the bacteria causing neonatal sepsis in our NICU were multi-drug-resistant organisms (MDROs). Only three of the 93 samples were not classified as MDROs, with two (2.2%) *Klebsiella pneumoniae* and one (1.1%) *Sphingomonas paucimobilis*.

*Klebsiella pneumoniae* is notorious for acquiring antibiotic resistance determinants, and it is classified as “critical” on the WHO global priority pathogen list. It is one of the ESKAPE pathogens (*Enterococcus faecium*, *Staphylococcus aureus*, *Klebsiella pneumoniae*, *Acinetobacter baumannii*, *Pseudomonas aeruginosa*, and *Enterobacter* spp.) that are primarily responsible for antibiotic resistance spreading in hospital-acquired infections [32]. *K. pneumoniae* is a major pathogen in both community-acquired and nosocomial neonatal infections, with case fatality rates ranging from 18% to 68%. Drug-resistant *K. pneumoniae* has emerged as a significant pathogen in recent years, with serious consequences due to limited antibiotic options, increased hospital expenditure, and poor neonatal outcomes [33]. *Klebsiella*'s presence in both the environment and the human body enables it to acquire a wide range of antibiotic resistance determinants. *K. pneumoniae* has nearly 400 antibiotic resistance genes, which is nearly double that of other pathogens. *K. pneumoniae* has a selective advantage because it lives in both the soil and the gut, which are both hotspots for the intergenus transfer of antibiotic resistance. The majority of antibiotic resistance determinants either originate in *K. pneumoniae* or are quickly acquired by this organism [32].

The use of a consistent gold standard for the diagnosis of neonatal septicemia as a key strength of this study lends credibility to our findings. This study also provides useful information that, for the first time, reveals the pathogen composition of neonatal sepsis in our hospital, which will be very useful in preventing and treating neonatal sepsis. Our variable scale was broad enough to describe the characteristics of the study population. But this study must be viewed with some limitations in mind. First, it was a single-center study with a relatively small sample size, which may lack the scientific precision or external validity required to aid in widespread changes in practice. Second, because it was done retrospectively, there may have been gaps in the data, one of which was that we could not give an analysis of molecular data because it was not routinely performed on each patient who underwent a culture examination. Third, we did not track long-term outcomes in neonatal sepsis patients. Long-term, multicenter, and prospective clinical studies are thus suggested for the future. Since the prevalence of sepsis in this study was influenced by nosocomial infection, it is crucial to follow the recommended infection control procedures, practice good hand hygiene, and have access to basic supplies and equipment, including sinks, running water, and disposables.

## 5. Conclusion

The most common microorganisms causing neonatal sepsis in our NICU were gram-negative bacteria, particularly *Klebsiella pneumoniae XDR*. Most of the study population was female and presented with EOS, being preterm with a low birth weight that was appropriate for gestational age. To prevent and reduce the incidence of sepsis, it is critical to follow the recommended infection-control protocols, practice excellent hand hygiene, and have access to essential supplies and equipment.

## Data Availability

The data used to support the findings of this study are included within the article.

## Abbreviations

AGA: Appropriate for gestational age
AST: Antimicrobial susceptibility testing
BAP: Blood agar plates
CONS: Coagulase-negative *Staphylococcus*
CRP: C-reactive protein
EOS: Early-onset sepsis
ESBL: Extended spectrum beta-lactamases
ESKAPE: *Enterococcus faecium*, *Staphylococcus aureus*, *Klebsiella pneumoniae*, *Acinetobacter baumannii*, *Pseudomonas aeruginosa*, and *Enterobacter* spp.
HALOS: Hospital-acquired late-onset sepsis
IAP: Intrapartum antibiotic prophylaxis
ICD: International Classification of Diseases
IgG: Immunoglobulin G
IV: Invasive ventilation
LBW: Low birth weight
LGA: Large for gestational age
LMICs: Low- and middle-income countries
LOS: Late-onset sepsis
MDR: Multidrug resistance
MDROs: Multi-drug-resistant organisms
mg/dL: Milligrams per deciliter
mL: Millilitre
MRSA: Methicillin-resistant *Staphylococcus aureus*
NICE: National Institute for Health and Care Excellence
NICU: Neonatal Intensive Care Unit
NIV: Noninvasive ventilation
PDA: Patent ductus arteriosus
ProM: Premature rupture of the membrane
sFcRIII: Soluble FcRIII receptor
SGA: Small for gestational age
SPSS: Statistical Package for the Social Sciences
WHO: World Health Organization
XDR: Extensively drug-resistant.
